# *De novo* Transcriptome Characterization of *Rhodomyrtus tomentosa* Leaves and Identification of Genes Involved in α/β-Pinene and β-Caryophyllene Biosynthesis

**DOI:** 10.3389/fpls.2018.01231

**Published:** 2018-08-24

**Authors:** Si-Mei He, Xiao Wang, Sheng-Chao Yang, Yang Dong, Qi-Ming Zhao, Jian-Li Yang, Kun Cong, Jia-Jin Zhang, Guang-Hui Zhang, Ying Wang, Wei Fan

**Affiliations:** ^1^State Key Laboratory of Conservation and Utilization of Bio-Resources in Yunnan, The Key Laboratory of Medicinal Plant Biology of Yunnan Province, National and Local Joint Engineering Research Center on Germplasm Innovation and Utilization of Chinese Medicinal Materials in Southwest China, Yunnan Agricultural University, Kunming, China; ^2^State Key Laboratory of Genetic Resources and Evolution, Kunming Institute of Zoology, Chinese Academy of Sciences, Kunming, China; ^3^Kunming College of Life Science, University of Chinese Academy of Sciences, Kunming, China; ^4^Province Key Laboratory, Biological Big Data College, Yunnan Agricultural University, Kunming, China; ^5^State Key Laboratory of Plant Physiology and Biochemistry, College of Life Sciences, Zhejiang University, Hangzhou, China; ^6^Key Laboratory of South China Agricultural Plant Molecular Analysis and Genetic Improvement, South China Botanical Garden, Chinese Academy of Sciences, Guangzhou, China

**Keywords:** *Rhodomyrtus tomentosa*, transcriptome, terpenoids, biosynthesis, terpene synthases

## Abstract

Plant-derived terpenes are effective in treating chronic dysentery, rheumatism, hepatitis, and hyperlipemia. Thus, understanding the molecular basis of terpene biosynthesis in some terpene-abundant Chinese medicinal plants is of great importance. Abundant in mono- and sesqui-terpenes, *Rhodomyrtus tomentosa* (Ait.) Hassk, an evergreen shrub belonging to the family Myrtaceae, is widely used as a traditional Chinese medicine. In this study, (+)-α-pinene and β-caryophyllene were detected to be the two major components in the leaves of *R. tomentosa*, in which (+)-α-pinene is higher in the young leaves than in the mature leaves, whereas the distribution of β-caryophyllene is opposite. Genome-wide transcriptome analysis of leaves identified 138 unigenes potentially involved in terpenoid biosynthesis. By integrating known biosynthetic pathways for terpenoids, 7 candidate genes encoding terpene synthase (RtTPS1-7) that potentially catalyze the last step in pinene and caryophyllene biosynthesis were further characterized. Sequence alignment analysis showed that RtTPS1, RtTPS3 and RtTPS4 do not contain typical N-terminal transit peptides (62–64aa), thus probably producing multiple isomers and enantiomers by terpenoid isomerization. Further enzyme activity *in vitro* confirmed that RtTPS1-4 mainly produce (+)-α-pinene and (+)-β-pinene, as well as small amounts of (−)-α-pinene and (−)-β-pinene with GPP, while RtTPS1 and RtTPS3 are also active with FPP, producing β-caryophyllene, along with a smaller amount of α-humulene. Our results deepen the understanding of molecular mechanisms of terpenes biosynthesis in Myrtaceae.

## Introduction

Terpenoids are large and diverse secondary metabolites, which play crucial roles in plant growth and development, pollinator attraction, and defense response (Bakkali et al., 2008). Terpenoids can be divided into seven categories according to the number of isoprene units that compose their backbone structure (Yazaki, 2006): mono-(C_10_), sesqui-(C_15_), di-(C_20_), sester-(C_25_), tri-(C_30_), tetra-(C_40_), and polyterpenes (C_n_) (Galata et al., 2014). In general, terpenoids are abundant in essential oils (EOs) and resins across a variety of plant species, and they are also emitted from foliage and flowers (Ahmad and Misra, 1994; Brown et al., 2003; McKay et al., 2003; Ruan et al., 2016).

**FIGURE 1:**
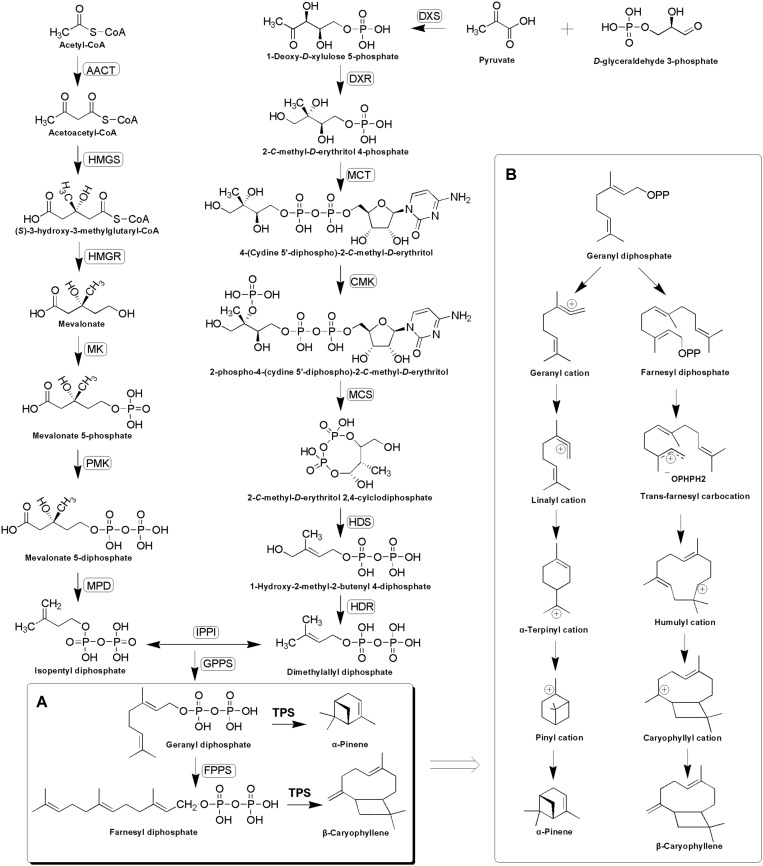
Putative α-pinene and β-caryophyllene biosynthesis pathway **(A)** and complex chemical mechanisms involving ionizations, isomerizations, cyclizations, and rearrangements catalyzed by TPSs **(B)**. The enzymes found in this study are boxed. AACT, acetyl-CoA-acetyltransferase; HMGS, hydroxymethylglutaryl-CoA synthase; HMGR, hydroxymethylglutaryl-CoA reductase; MK, mevalonate kinase; PMK, phosphomevalonate kinase; MPD, diphosphomevalonate decarboxylase; DXS, 1-deoxy-D-xylulose-5-phosphate synthase; DXR, 1-deoxy-D-xylulose-5-phosphate reductoisomerase; MCT, 2-*C*-methyl-D-erythritol 4-phosphate cytidylyltransferase; CMK, 4-diphosphocytidyl-2-*C*-methyl-D-erythritol kinase; MCS, 2-*C*-methyl-D-erythritol 2,4-cyclodiphosphate synthase; HDS, (E)-4-hydroxy-3-methylbut-2-enyl-diphosphate synthase; HDR, 4-hydroxy-3-methylbut-2-enyl diphosphate reductase; IPPI, isopentenyl-diphosphate delta-isomerase; GPPS, geranyl diphosphate synthase; FPPS, farnesyl diphosphate synthase.

Terpenoids are derived from two five-carbon compounds isopentenyl pyrophosphates (IPP) and its allylic isomer dimethylallyl pyrophosphate (DMAPP) via two alternative pathways: the cytosolic mevalonate (MVA) pathway and the plastidial methylerythritol phosphate (MEP) pathway (Nagegowda, 2010; Singh and Sharma, 2015). Condensation of one DMAPP and one IPP catalyzed by geranyl diphosphate synthase (GPPS) leads to the formation of geranyl diphosphate (GPP) in the plastids, whereas the condensation of one DMAPP and two IPP molecules catalyzed by farnesyl diphosphate synthase (FPPS) leads to the formation of farnesyl diphosphate (FPP) in the cytosol (Despinasse et al., 2017). GPP and FPP serve as substrates for terpene synthases (TPSs) for synthesizing mono- and sesqui-terpenes, respectively (Yahyaa et al., 2015; Despinasse et al., 2017), during which the synthesis of monoterpenes is initiated by GPP dephosphorylation and ionization to geranyl carbocation, while the synthesis of sesquiterpene starts with FPP ionization to a farnesyl cation (Degenhardt et al., 2009; Huang et al., 2010). This is then followed by a series of complex chemical mechanisms involving isomerizations, cyclizations, and rearrangements catalyzed by TPSs, which finally generate structurally diverse terpenoids (Keszei et al., 2010; Koo et al., 2016; Piechulla et al., 2016) (**Figure 1**).

Efforts have previously been made to characterize TPSs from a variety of plant species, including *Arabidopsis thaliana* (Tholl et al., 2005), *Cucumis melo* (Portnoy et al., 2008), *Gossypium hirsutum* (Huang et al., 2013), *Solanum lycopersicum* (Falara et al., 2011), *Vitis vinifera* (Martin et al., 2010), *Coriandrum sativum* (Galata et al., 2014), and *Thapsia garganica* (Pickel et al., 2012). Three genes, namely *AaTPS2*, *AaTPS5*, and *AaTPS6*, encoding monoterpene synthases were found to catalyze the formation of β-myrcene, camphene, and 1, 8-cineole, respectively, in *Artemisia annua* (Ruan et al., 2016). Ma et al. (2016) characterized a monoterpene synthase from *Paeonia lactiflora*, which produces α-pinene as its single product. In cotton, (*E*)-β-caryophyllene synthase and α/β-pinene synthase were found to be potentially involved in constitutive and herbivore-induced terpene formation (Huang et al., 2013). In addition, *OkBCS* encoding β-caryophyllene synthase from *Ocimum kilimandscharicum* Gürke was identified by means of transient silencing and overexpression, it was found to function in β-caryophyllene biosynthesis (Jayaramaiah et al., 2016). The identification of these genes and enzymes furthers our understanding of the catalytic mechanisms of TPS in plants.

The family Myrtaceae is one of the most significant essential oil-yielding plant families and it is known for high terpene concentrations in the foliage (Keszei et al., 2010). Despite an abundance of chemical information, the molecular mechanisms underlying terpene biosynthesis are poorly understood. Keszei et al. (2010) described 70 unique partial terpene synthase transcripts and 8 full-length cDNA clones from 21 myrtaceous species, first charactering a 1,8-cineole synthase from *Eucalyptus sideroxylon* and a caryophyllene synthase from *Eucalyptus dives* based on phylogenetic relationships and leaf oil composition. Recently, *Eugenia uniflora* was used to identify genes involved in the terpene biosynthesis pathway by high-throughput RNA sequencing, yielding several predicted candidate *TPSs* associated with mono-, sesqui-, and tri-terpenes biosynthesis (Guzman et al., 2014). However, functional characterization of related genes encoding TPS in Myrtaceous is still lacking. As an abundance and variety of terpenoids exist in Myrtaceae owing to the structural differences of the TPSs present (Keeling et al., 2008; Keszei et al., 2010), further research into terpene biosynthesis is required.

*Rhodomyrtus tomentosa* (Ait.) Hassk (Myrtaceae), widely distributed in East Asia and Southeast Asia, including Japan, Thailand, and southern China, is an evergreen shrub (Keszei et al., 2008; Saising et al., 2011). The stems, leaves, and fruits of *R. tomentosa* are widely used as a traditional medicine to treat chronic dysentery, rheumatism, hepatitis, and hyperlipemia by virtue of high content of mono- and sesqui-terpenes (Chen, 1984). However, a lack of genomic information hinders our understanding for terpenoid biosynthesis in this species. In this study, the main active components in the leaves of *R. tomentosa* were identified. 138 unigenes potentially involved in terpenoid biosynthesis though transcriptome analysis depicted a complete biosynthesis pathway for α-pinene and β-caryophyllene. Further enzyme activity *in vitro* confirmed some *TPS* genes function in α/β-pinene and β-caryophyllene biosynthesis.

## Results and Discussion

### Gas Chromatography–Mass Spectrometry Analysis of Terpenoids in *R. tomentosa* Leaves

Gas chromatography–mass spectrometry (GC–MS) technique was used to analyze volatile compound composition of the young and mature leaves in *R. tomentosa*. We identified four monoterpenes ((+)-α-pinene, (−)-α-pinene, (+)-β-pinene and (−)-β-pinene) and two sesquiterpenes (β-caryophyllene and α-humulene) by virtue of authentic standards and GC–MS database (**Table 1** and **Supplementary Figure S1**). Among them, (+)-α-pinene and β-caryophyllene were most abundant in the leaves, representing 88.43 and 6.68% of total terpenoids in the young leaves, and 85.42 and 10.24% in the mature leaves. With the exception of β-caryophyllene, other compounds were more abundant in the young leaves than in the mature leaves. These results suggest that young leaves are more efficient in terpenoids biosynthesis. Plant terpenoids can be divided into inducible and constitutive compounds. Inducible terpenes (e.g., β-ocimene) are synthesized *de novo* in response to herbivore damage (Paré and Tumlinson, 1997), whereas constitutive terpenes (e.g., α-pinene and β-caryophyllene) are stored in subepidermal pigment glands, and are immediately released upon herbivore feeding and leaf damage (Elzen et al., 1985; McCall et al., 1994; Röse et al., 1996; Paré and Tumlinson, 1997). Usually, glandular trichomes are more abundant in the young leaves, thus explaining why the young leaves of *R. tomentosa* possess more mono- and sesqui-terpenes.

**Table 1:** GC-MS analysis of the compounds in *R. tomentosa* leaves.

Peak	Retention time (min)		Compounds	Abundance(%)	
	Young leaves	Mature leaves	Young and Mature leaves	Young leaves	Mature leaves
1	12.582	12.607	(−)-α-pinene	2.61 ± 1.3	2.42 ± 2.5
2	12.651	12.677	(+)-α-pinene	88.43 ± 1.1	85.42 ± 1.8
3	14.123	14.145	(+)-β- pinene	1.14 ± 0.4	0.91 ± 0.2
4	14.340	14.364	(−)-β- pinene	0.42 ± 0.3	0.36 ± 0.7
5	26.497	26.520	β-caryophyllene	6.68 ± 0.9	10.24 ± 1.4
6	27.420	27.447	α-humulene	0.72 ± 0.6	0.64 ± 0.2

### Illumina Sequencing, *de novo* Assembly and Function Annotation

In order to further understand the molecular mechanism of terpenoid biosynthesis in *R. tomentosa*, a comprehensive transcriptome in *R. tomentosa* leaves were performed, which generated a total of 202,153,314 clean reads (total length of 30,431,756,559; 30.4 Gb) after removing the adaptor sequences, ambiguous reads, and low-quality reads (Q20 < 20) (**Supplementary Table S1**). The Q20 (sequencing error rate < 1%) and GC percentages were 97.50 and 48.45%, 97.47 and 48.96%, 95.92 and 50.17%, respectively (**Supplementary Table S1**). The high-quality reads were deposited in the NCBI SRA database (accession number: SRP132648).

Considering unavailable reference genome for *R. tomentosa*, Trinity (Trinityrnaseq_r20131110) were used to *de novo* assemble all of the clean reads (Grabherr et al., 2011). A total of 146,480 contigs ranging from 201 to 21,224 bp, with a mean length of 1,379 bp and an N50 length of 2,161 bp were assemble (**Supplementary Table S1**). Overall, these transcripts represented 83,175 unigenes with an average length of 888 bp and an N50 length of 1,702 bp, among which 53,334 coding DNA sequences (CDS) were detected (**Supplementary Table S1**). By searching against five public protein databases, a total of 53,742 unigenes (64.61%) were annotated. Among them, the unigenes matched to NCBI non-redundant protein sequences (NR), SWISS-PROT, eukaryotic ortholog groups (KOG), kyoto encyclopedia of genes and genomes (KEGG) and gene ontology (GO) databases were 49,545 (59.57%), 42,547(51.15%), 35,016 (42.09%), 26,096 (31.37%) and 8,902 (10.7%), respectively (**Supplementary Table S2**).

The KEGG pathway-based analysis could help in understanding the biological functions of genes. A total of 12,533 unigenes were annotated and assigned to 132 KEGG pathways. The category with the largest number of unigenes was metabolism, which included amino acid metabolism (1,991 unigenes, 16.41%), biosynthesis of other secondary metabolites (710, 5.85%), carbohydrate metabolism (3,683, 30,36%), energy metabolism (2,016, 16.62%), glycan biosynthesis and metabolism (229, 1.89%), lipid metabolism (1,225, 10.10%), metabolism of cofactors and vitamins (672, 5.54%), metabolism of other amino acids (618, 5.09%), metabolism of terpenoids and polyketides (439, 3.62%), and nucleotide metabolism (549, 4.53%) (**Supplementary Figure S2A**). With regards to terpenoid and polyketide metabolism, the most represented category was terpenoid backbone biosynthesis (168, 38.27%), followed by carotenoid biosynthesis (92, 20.96%); limonene and pinene degradation (42, 9.57%); diterpenoid biosynthesis (38, 8.66%); brassinosteroid biosynthesis (31, 7.06%); sesquiterpenoid and triterpenoid biosynthesis (28, 6.38%); monoterpenoid biosynthesis (22, 5.01%); and zeatin biosynthesis (18, 4.10%) (**Supplementary Figure S2B**).

### Candidate Genes Encoding Enzymes Involved in Terpenoid Biosynthesis

According to the GC–MS results, α/β-pinene, β-caryophyllene and α-humulene constitute the major active components in *R. tomentosa*. Among them, (+)-α-pinene and β-caryophyllene were most abundant in the leaves (**Table 1**). α-pinene, a monocyclic monoterpene, is an important raw material in the spice synthesis, while β-caryophyllene has antimicrobial, antioxidant, and anticarcinogenic properties, enhanced skin penetration properties, and is also an approved food additive (Cornwell and Barry, 1994; Kubo et al., 1996; Lourens et al., 2004; Singh et al., 2006, 2014; Huang et al., 2012). Therefore, biosynthesis pathway of α/β-pinene and β-caryophyllene were focused. The synthesis of IPP and its isomer DMAPP known as GPP and FPP precursors involves multistep enzyme catalytic reaction in the MVA and MEP pathways. Interestingly, 138 potential genes encoding these enzymes were identified by blasting the known protein database, including acetyl-CoA-acetyltransferase (AACT); hydroxymethylglutaryl-CoA synthase (HMGS); hydroxymethylglutaryl-CoA reductase (HMGR); mevalonate kinase (MK); phosphomevalonate kinase (PMK); diphosphomevalonate decarboxylase (MPD); 1-deoxy-D-xylulose-5-phosphate synthase (DXS); 1-deoxy-D-xylulose-5-phosphate reductoisomerase (DXR); 2-*C*-methyl-D-erythritol 4-phosphate cytidylyltransferase (MCT); 4-diphosphocytidyl-2-*C*-methyl-*D*-erythritol kinase (CMK); 2-*C*-methyl-D-erythritol 2,4-cyclodiphosphate synthase (MCS); (*E*)-4-hydroxy-3-methylbut-2-enyl-diphosphate synthase (HDS); 4-hydroxy-3-methylbut-2-enyl diphosphate reductase (HDR); isopentenyl-diphosphate delta-isomerase (IPPI) (**Table 2**, **Figure 1**, and **Supplementary Table S3**). Finally, IPP and isomer DMAPP synthesize GPP and FPP by geranyl diphosphate synthase (GPPS) and farnesyl diphosphate synthase (FPPS), respectively, which are then diverted to different mono- (e.g., α-pinene) and sesqui-terpenes (e.g., β-caryophyllene), respectively, after a single step enzymatic reaction mediated by TPS (Keszei et al., 2010; Koo et al., 2016; Piechulla et al., 2016) (**Figure 1**). Therefore, identifying the function of TPS enzymes in the last step are important for understanding the molecular mechanism of terpenoid synthesis in *R. tomentosa*.

**Table 2:** Transcripts involved in terpenoid biosynthesis in *R. tomentosa* leaves.

Gene name	EC number	Unigene numbers
AACT, acetyl-CoA-acetyltransferase	2.3.1.9	12
HMGS, hydroxymethylglutaryl-CoA synthase	2.3.3.10	9
HMGR, hydroxymethylglutaryl-CoA reductase	1.1.1.34	10
MK, mevalonate kinase	2.7.1.36	3
PMK, phosphomevalonate kinase	2.7.4.2	2
MPD, diphosphomevalonate decarboxylase	4.1.1.33	2
DXS, 1-deoxy-D-xylulose-5-phosphate synthase	2.2.1.7	10
DXR, 1-deoxy-D-xylulose-5-phosphate reductoisomerase	1.1.1.267	4
MCT, 2-*C*-methyl-D-erythritol 4-phosphate cytidylyltransferase	2.7.7.60	1
CMK, 4-diphosphocytidyl-2-*C*-methyl-D-erythritol kinase	2.7.1.148	2
MCS, 2-*C*-methyl-D-erythritol 2,4-cyclodiphosphate synthase	4.6.1.12	7
HDS, (*E*)-4-hydroxy-3-methylbut-2-enyl-diphosphate synthase	1.17.7.1	15
HDR, 4-hydroxy-3-methylbut-2-enyl diphosphate reductase	1.17.1.2	14
IPPI, isopentenyl-diphosphate delta-isomerase	5.3.3.2	12
GPPS, geranyl diphosphate synthase	2.5.1.1	29
FPPS, farnesyl diphosphate synthase	2.5.1.10	6

**FIGURE 2:**
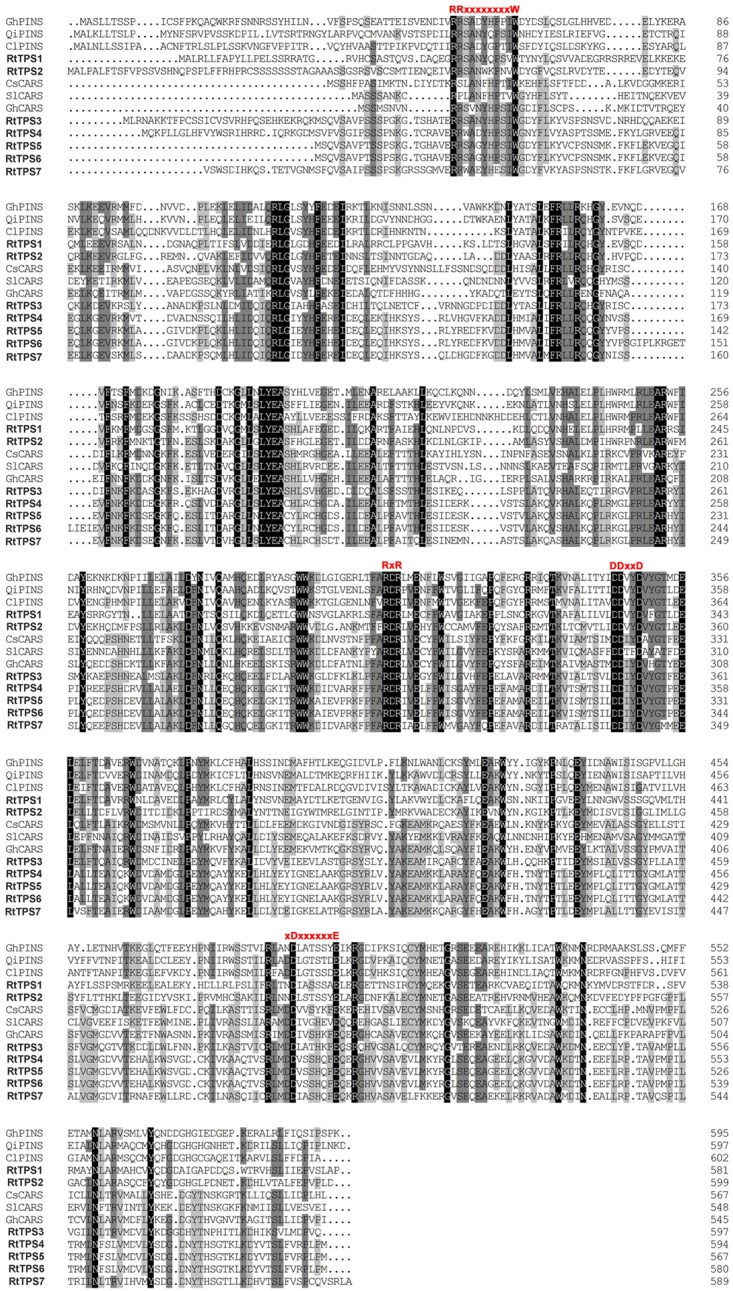
Alignment of amino acid sequences of *R. tomentosa* TPSs and orthologous proteins from other plant species, including *Gossypium hirsutum* (GhPINS, AGX84977), *Quercus ilex* (QiPINS, AM283099), *Citrus limon* (ClPINS, AAM53945), *Cucumis sativus* (CsCARS, AAU05952), *Solanum lycopersicum* (SlCARS, ADD96698), and *G. hirsutum* (GhCARS, AFQ23183). Different conserved domains are indicated in red.

**FIGURE 3:**
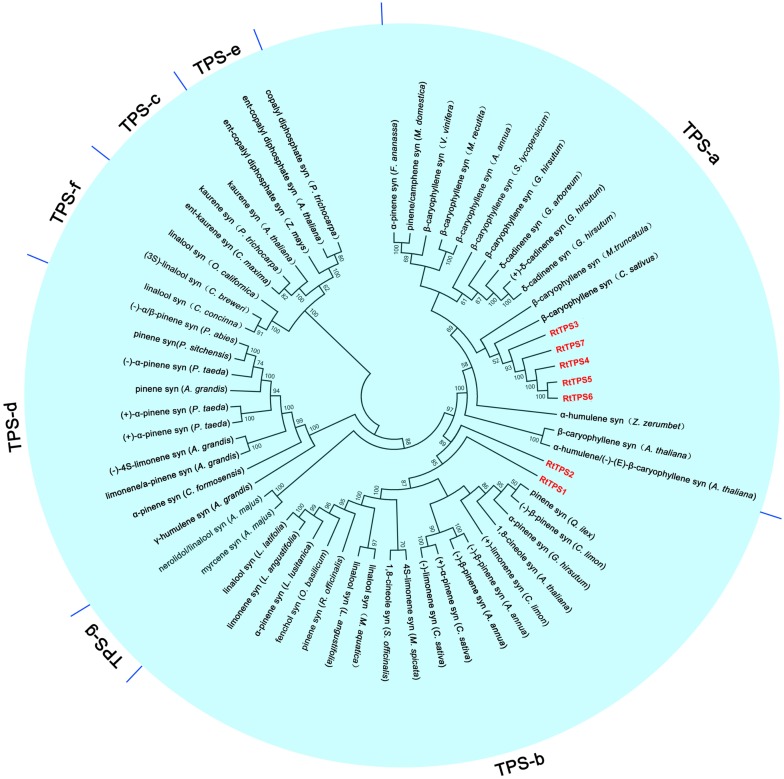
Phylogenetic analysis of TPSs. The phylogenetic tree was constructed based on the deduced amino acid sequences for the *R. tomentosa* TPSs (red letters) and other plant TPSs. GenBank accession numbers for the sequences used are provided in **Supplementary Table S5**.

A total of seven unigenes encoding TPS were identified from the RNA-seq data. The amino acid comparison showed 63–65% similarity and 41–42% identity among RtTPS1 to 4, while RtTPS4-7 have high similarity with 92% and similarity with 91%. Compared with other species, RtTPS1 and RtTPS2 are more homologous to PINS from *G. hirsutum* and *Quercus ilex*, whereas RtTPS3 to 7 shares high homology with CARS from *Cucumis sativus* and *S. lycopersicum* (**Supplementary Table S4**). Alignment of the amino acid sequences indicated that RtTPS1 to 7 contain highly conserved motifs along with other known TPSs (Fischbach et al., 2001; Lücker et al., 2002; Mercke et al., 2004; Schilmiller et al., 2010; Huang et al., 2013; Yang et al., 2013) (**Figure 2**). An aspartate-rich motif DDxxD is crucial for substrate binding, while DDxxxxxxE functions in metal cofactor binding (Köllner et al., 2004). Both are required for GPP and FPP ionization (Rynkiewicz et al., 2001; Degenhardt et al., 2009). It was reported that RxR motif located 35 amino acids upstream of DDxxD motif has been implicitly involved in the complexation of diphosphate group after substrate ionization, while RR(X)8W motif present in the N-terminal region might participate in the isomerization of the substrate and is characteristic of the majority of TPS-a and TPS-b subfamilies (Starks et al., 1997; Bohlmann et al., 1998; Williams et al., 1998). Phylogenetic analysis demonstrated that RtTPS1-2 and RtTPS3-7 clustered closely with their orthologs from other plants, belonging to the TPS-b and TPS-a subfamily, respectively (**Figure 3**).

### Enzyme Activity of RtTPS1-4

**FIGURE 4:**
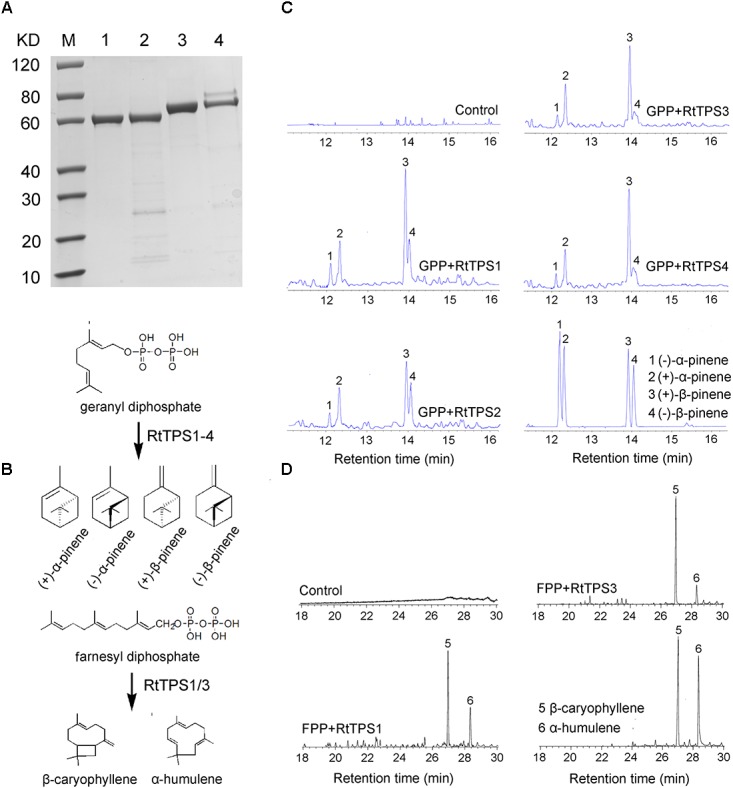
Enzymatic characterization of recombinant protein RtTPS1-4. **(A)** His-tag purified recombinant RtTPS1-4 on 12% SDS–PAGE gel, Lane M: Protein Marker, Lane 1: purified RtTPS1, Lane 2: purified RtTPS2, Lane 3: purified RtTPS3, Lane 4: purified RtTPS4; **(B)** verified reaction equation; pentane extracts of RtTPS1-4 after incubation with GPP **(C)** and FPP **(D)**, respectively, were detected by GC–MS. pET30a empty vector was used as control. Products were identified using authentic standards. 1, (+)-α-pinene; 2, (−)-α-pinene; 3, (+)-β-pinene; 4, (−)-β-pinene; 5, β-caryophyllene; 6, α-humulene.

According to phylogenetic tree analysis, RtTPS1 and RtTPS2 are clustered in the branch of pinene synthase, while RtTPS3 to 7 are clustered in the branch of β-caryophyllene synthase (**Figure 3**). Therefore, *RtTPS 1-4* were selected to perform enzyme activity *in vitro* by heterologous expression and GC-MS detection. The results showed that RtTPS1-4 produce mainly (+)-α-pinene and (+)-β-pinene, as well as small amounts of (−)-α-pinene and (−)-β-pinene with GPP, while RtTPS1 and RtTPS3 are also active with FPP, producing β-caryophyllene, along with a smaller amount of α-humulene (**Figure 4**).

RtTPS 1-4 contain all the conserved domains of the TPS family, including the RR(P)X_8_W, RXR, and DDXXD (X is any amino acid) motifs, and absolutely conserved arginine, cysteine and histidine residues in active-site (Bohlmann et al., 1998). However, our results indicated that RtTPS1, RtTPS3-4 can catalyze the formation of α/β-pinene, β-caryophyllene and α-humulene (**Figure 4**). The amino acid number of RtTPS1, RtTPS3-4 at upstream of conserved RRX8W motif is 38, 52, and 48, respectively, which result in an incomplete plastidial targeting sequence (62–64 aa) (**Figure 2**). Bohlmann et al. (1998) suggested that short N-terminal sequence may play a role in the isomerization step of the terpenoid cyclization reaction, thus producing multiple pinene isomers and enantiomers. In addition, differences in amino acids between loop and helix in domains may also lead to diverse products. Phillips et al. (2003) reported that these differences could conceivably determine substrate folding, thereby providing a stereochemical switch. Domain swapping and directed mutagenesis in (−)-(4*S*)-limonene synthase (LS) and (−)-(4*S*)-limonene/(−)-(1*S*, 5*S*)-α-pinene synthase (LPS) from *Abies grandis* suggested that amino acids in the predicted D through F helix regions are critical for product determination (Katoh et al., 2004).

The chiral analysis showed that RtTPS1-4 can produce more (+)-α-pinene and (+)-β-pinene than (−)-α-pinene and (−)-β-pinene, which may be related to the pH conditions of enzyme activity. TPSs preferentially form (+)-pinenes under acidic conditions, whereas (−)-pinenes is easily formed under alkaline conditions (Phillips et al., 1999). A pinene synthase from *A. grandis* that produces both (−)-α- and (−)-β-pinene in pH 7.5 assay buffer (Bohlmann et al., 1997). In our experiments, the neutral conditions of pH 7 may contribute to (+)-/(−)-pinenes. The results of enzyme activity are consistent with those detected in volatile oil from leaves.

Interestingly, RtTPS1 and RtTPS3 were also able to catalyze FPP to produce β-caryophyllene (65.71% for RtTPS1 and 93.05% for RtTPS3) and α-humulene (34.29% for RtTPS1 and 6.95% for RtTPS3) (**Figure 4B** and **Supplementary Table S7**). In previous reports, *G. hirsutum* TPS1 (GhTPS1) and OkBCS from *O. kilimandscharicum* were also reported to produce mainly β-caryophyllene and smaller amounts of α-humulene (Huang et al., 2013; Jayaramaiah et al., 2016). Moreover, the ratio of two substances catalyzed by OkBCS is similar to our results (Jayaramaiah et al., 2016). In addition, CsTPS4FN, CsTPS5FN and CsTPS9FN in *Cannabis sativa* could also collectively catalyze GPP and FPP. Especially, CsTPS5FN was an unusual TPS-b member lacking a N-terminal plastidial targeting sequence, which was able to produce sesquiterpenes when incubated with FPP (Booth et al., 2017). It is well known that monoterpenes are synthesized in plastids, while sesquiterpenes are synthesized in cytosol (Jayaramaiah et al., 2016; Ruan et al., 2016). TPS1 and TPS3 produced monoterpenes and sesquiterpenes simultaneously, but they did not have N-terminal plastid-targeting sequences (**Figures 2**, **4**). This requires the transport of IPP/DMAPP (GPP and FPP precursor) between plastid and cytosol, as occurs in hop trichomes and snapdragon flowers (Dudareva et al., 2005; Wang et al., 2008). Overall, diverse structure and extensive catalytic activities of RtTPS1-4 suggested divergent and convergent functions of *R. tomentosa* TPSs in terpene biosynthesis.

### Expression Patterns of RtTPS1-7

**FIGURE 5:**
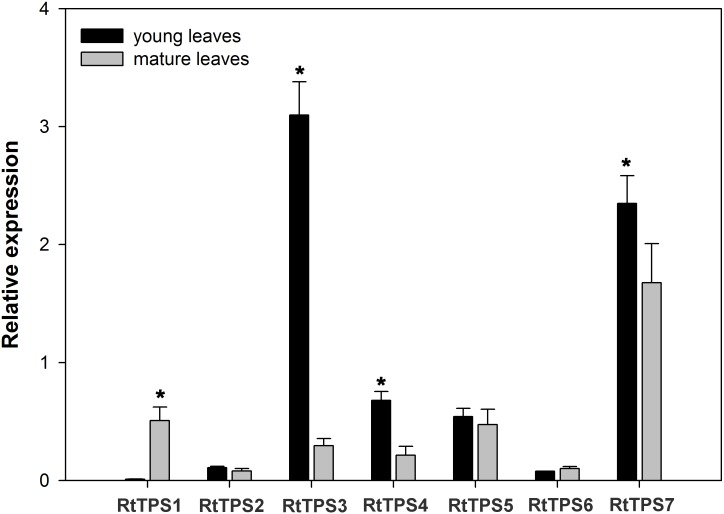
Expression patterns of *R. tomentosa* TPSs. The transcripts were analyzed by qRT-PCR, with actin as an internal standard. Error bars indicate the standard deviation (SD) of three biological replicates. The significant differences between samples were statistically evaluated by Student’s *t*-test method. ^∗^*P* < 0.05.

To study the TPS gene expression in the young and mature leaves of *R. tomentosa*, we measured the accumulation of *RtTPS 1-7* transcripts using reverse transcription quantitative real-time PCR (qRT-PCR) (**Figure 5**). The expression levels of *RtTPS3, RtTPS4* and *RtTPS7* had significantly expressed levels in the young leaves than in the mature leaves, while *RtTPS1* were more highly expressed in the mature leaves than in the young leaves, and *RtTPS2, RtTPS5* and *RtTPS6* showed similar expression in the two tissues. These results suggest that candidate TPS genes involved in pinene and caryophyllene were differentially regulated at transcriptional level. In terms of the four candidate genes encoding to RtTPS1-4, the expression levels of RtTPS3 and RtTPS4 in the young leaves were higher than that of RtTPS1 and RtTPS2, contributing probably to more pinene biosynthesis in the young leaves. Similarly, high expression level of RtTPS3 may also contribute to high biosynthesis of caryophyllene in the young leaves. However, the expression levels of these genes did not correlate well with the synthetic level in the old leaves, suggesting that other TPS transcripts which failed to identify in our study can lead to accumulation of pinene and caryophyllene in the old leaves, since transcriptome only represents a set of transcripts of a period.

## Conclusion

This was the first attempt to elucidate the *R. tomentosa* transcriptome using Illumina next-generation sequencing and *de novo* assembly. A total of 138 unigenes involved in the biosynthesis of the terpenoids were identified in *R. tomentosa*. Based on GC-MS and transcriptome results, a complete biosynthesis pathway for α-pinene and β-caryophyllene were depicted. Enzyme activity assay *in vitro* confirmed RtTPS1-4 function in biosynthesis of α/β-pinene and β-caryophyllene in *R. tomentosa*, suggesting overlapped and divergent functions of TPS in plant species.

## Materials and Methods

### Plant Materials and GC-MS Analysis

The 3-year old plants growing in South China Botanical Garden Chinese Academy of Sciences in Guangzhou, China were collected. The leaves of 5 independent plants were one biological replication, and three biological replications were performed. The young leaves are the opposite leaves of the first node on the branches, showing small leaf area and light color, while the old leaves are opposite leaves of eighth node on the branches, showing large leaf area and leathery green. Fresh leaves of *R. tomentosa* (50.0 g) were extracted with 500mL of deionized water and 30mL of dichloromethane using Simultaneous Distillation-Extraction for 3 h. GC–MS analysis was performed in an Agilent 7890 GC system coupled with a 5975 MS detector (Agilent Technologies, United States). The essential oil, diluted 10 times using dichloromethane, was added as an internal standard. One μL was injected in split mode in an HP5-MS column (30 m × 250 μm × 0.25 μm film thickness). The temperature program included: an initial oven temperature of 40°C (1 min hold), followed by a two-step temperature increase, first to 130°C (at a rate of 4°C min^−1^, 5 min hold) and then to 250°C (at a rate of 10°C min^−1^, 5 min hold). MS conditions were: ionization mode: El, electron energy 70 eV; interface temperature: 280°C, ion source temperature: 230°C, quadropole temperature 150°C; carrier gas (helium) at a flow rate of 1.2 ml min^−1^; mass range 35–650 m/z. To separate α-pinene and β-pinene enantiomers, A GC model 5890II (Hewlett-Packard, Palo Alto, CA, United States) equipped with a CycloSil-B fused silica capillary column (Agilent J&W Scientific, i.d. 0.25 mm, 30 m, film thickness = 0.25 μm) and a flame ionization detection (FID) was used for measurement routine analysis of volatiles. The helium carrier gas flow rate was 1.0 mL/min at a split ratio of 50:1. The injector temperature was 250°C and the detector temperature was 340°C, respectively. The oven temperature was programmed from 55°C (held for 1 min) to 180°C at 5°C/min and then held constant for 15 min. Compounds were identified by blasting against NIST 05 (National Institute of Standards and Technology) libraries and their retention times and mass spectra with those of authentic standards (Sigma-Aldrich, Oakville, ON, Canada). The experiment was performed with three biological and technical replications.

### RNA Isolation and Solexa Sequencing

Total RNA of leaves were extracted from three independent plants using a TRIzol reagent (Invitrogen, United States) and digested with RNase free DNAase I (Qiagen, Germany). The cDNA libraries were constructed following the Illumina manufacturer’s instructions. In brief, the polyA^+^ RNA was purified from total RNA using Oligo(dT) magnetic beads and broken into short fragments using divalent cations at 94°C for 5 min. Using these short fragments as templates, random hexamer-primer was used to synthesize the first-strand cDNA, followed by the synthesis of second-strand cDNA using DNA polymerase I and RNase H. Short fragments were purified with a QiaQuick PCR Extraction Kit (Qiagen) and ligated to sequencing adapters. The products were amplified by PCR to create cDNA libraries. The cDNA libraries were sequenced using Illumina HiseqTM 2000 system.

### Sequence Assembly and Annotation

The sequencing-received raw image data were transformed by base calling into raw reads. Reads were assembled using Trinity software (Grabherr et al., 2011). The longest assembled sequences were referred to as contigs. Reads were then mapped back to contigs with paired-end reads to detect contigs from the same transcript and the distances between these contigs. N was used to connect each two contigs to represent unknown sequences, and then for Scaffold. Finally, sequences were obtained that lacked N and could not be extended on either end, and were defined as unigenes. The unigene sequences were aligned by BLASTx to various databases including the NR database^1^, SWISS-PROT^2^, KEGG^3^ (Kanehisa et al., 2006), and KOG database^4^ (Tatusov et al., 1997) using BLAST (*E*-value < 1E^−5^).

### Phylogenetic Analysis

Phylogenetic analysis was performed based on the deduced amino acid sequences of TPSs from *R. tomentosa* and other plants. All of the full-length protein sequence of TPSs was assembled using Clustal X 2.0 software, and created a bootstrap neighbor-joining evolutionary tree by MEGA 5.0 software with 1000 bootstrap replicates. The scale represents 0.1 amino acid substitutions per site.

### Recombinant Protein Purification and Enzyme Activity Assay

Full-length cDNAs of *TPSs* including *RtTPS1*, *RtTPS2*, *RtTPS3* and *RtTPS4* were PCR amplified using primers (**Supplementary Table S6**), and ligated into the pET*30a* vector. The constructed vector was introduced into the *Escherichia coli* strain BL21 (DE3) for protein expression. The recombinant protein was first induced with IPTG at 15°C for 16 h. Then, the cells were harvested and resuspended with binding buffer, (20 mM sodium phosphate, 0.5 M sodium chloride, and 40 mM imidazole pH 7.4). The recombinant enzyme was purified by Ni-IDA-Sepharose CL-6B (Spectrum Chemical Manufacturing, United States) according to the manufacturer’s instructions after renaturation by 2M urea. The purity of the His-tagged protein was determined by SDS-PAGE followed by Coomassie Brilliant Blue staining.

Enzyme activity assays were performed in a volume of 500 μl reaction buffer (25 mM HEPES, pH 7.0, 10 mM MgCl_2_, 5 mM dithiothreitol), containing 10 mM substrate (GPP and FPP, respectively) and 10 μg protein. After incubating for 30 min at 30°C, the reaction mixture was extracted with 500 μl pentane, and 1 μl was subjected to analysis by GC-MS and GC-FID as described above.

### qRT-PCR Analysis

Total RNA was isolated from young and mature leaves of *R. tomentosa* using a Trizol Kit (Promega, United States). First-strand cDNA was synthesized from 2 μg of purified RNA using HiScript QRT SuperMix for qPCR (Vazyme, Nanjing, China). Two microliter (100 ng μL^−1^) of cDNA in 20 μL solution systems was used for gene expression performed with SYBR Premix Ex Taq (Takara) on a Roche LightCycler 2.0 system (Roche Applied Science, Branford, CT, United States). The primers of genes were listed in **Supplementary Table S8**, and the PCR amplification conditions were as follows: 94°C for 5 min; 40 cycles of 95°C for 20 s, 55°C for 20 s, and 72°C for 30 s. For each gene, expression data were normalized with expression level of actin gene and calculated by 2^−ΔΔC_t_^ method. The experiment was carried out three biological and technical replications. The significant differences between samples were statistically evaluated by Student’s *t*-test method.

## Author Contributions

WF and YW conceived the study. S-MH and S-CY designed the experiments. S-MH, XW, Q-MZ, and KC performed the experiments. J-LY performed the chiral analysis of leaf volatiles and enzymatic products. YD and J-JZ analyzed the data. S-MH and WF wrote the manuscript. All authors read and approved the final manuscript.

## Conflict of Interest Statement

The authors declare that the research was conducted in the absence of any commercial or financial relationships that could be construed as a potential conflict of interest.

## Footnotes

1 http://www.ncbi.nlm.nih.gov/

2 http://www.expasy.ch/sprot/

3 http://www.genome.jp/kegg/

4 http://www.ncbi.nlm.nih.gov/COG
