# Supplementary material for: De novo Transcriptome Characterization of Rhodomyrtus tomentosa Leaves and Identification of Genes Involved in α/β-Pinene and β-Caryophyllene Biosynthesis
Source: Front Plant Sci. 2018 Aug 24;9:1231. doi: 10.3389/fpls.2018.01231 (PMC6117411; doi:10.3389/fpls.2018.01231)
Supplement: Supplementary file 1 [file Data_Sheet_1.DOC]

**Supplementary information for following article**

***De Novo* transcriptome characterization of *Rhodomyrtus tomentosa* leaves and identification of genes involved in α/β-pinene and β-caryophyllene biosynthesis**

Si-Mei He^1†^, Xiao Wang^2,5†^, Sheng-Chao Yang^1^, Yang Dong^3^, Qi-Ming Zhao^1^, Jian-Li Yang^4^, Kun Cong^1^, Jia-Jin Zhang^1^, Guang-Hui Zhang^1^, Ying Wang^6*^, Wei Fan^1*^

^1^ State Key Laboratory of Conservation and Utilization of Bio-resources in Yunnan, The Key Laboratory of Medicinal Plant Biology of Yunnan Province, National& Local Joint Engineering Research Center on Germplasm Innovation & Utilization of Chinese Medicinal Materials in Southwest China, Yunnan Agricultural University, Kunming, 650201, China.

^2^ State Key Laboratory of Genetic Resources and Evolution, Kunming Institute of Zoology, Chinese Academy of Sciences, Kunming 650201, China.

^3^ Province Key Laboratory, Biological Big Data College, Yunnan Agricultural University, Kunming 650201, China.

^4^ State Key Laboratory of Plant Physiology and Biochemistry, College of Life Sciences, Zhejiang University, Hangzhou 310058, China.

^5^ Kunming College of Life Science, University of Chinese Academy of Sciences, Kunming 650204, China.

^6^ Key Laboratory of South China Agricultural Plant Molecular Analysis and Genetic Improvement, South China Botanical Garden, Chinese Academy of Sciences, Guangzhou 510650, China.

^†^These authors have contributed equally to this work

***Corresponding authors:** Ying Wang, Wei Fan

**Telephone number:** +86-871-65228927

**Fax number:** +86-871-65227712

**E-mail:** yingwang@scib.ac.cn, fanwei1128@aliyun.com

**
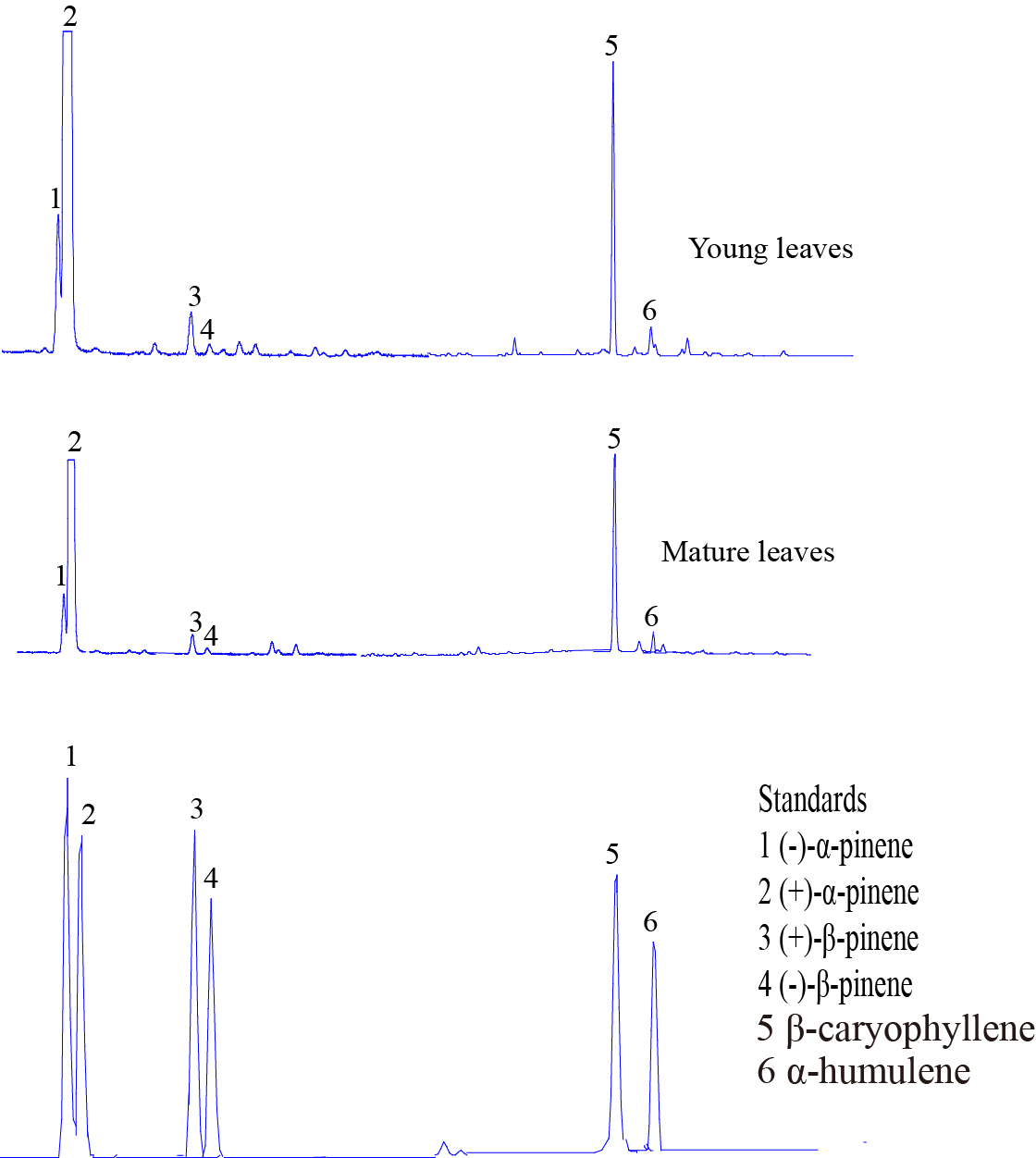
**

**Supplemental Fig. 1. GC−MS and GC-FID results indicating the major terpenoids produced by *R. tomentosa.*** GC−MS and GC-FID analysis of major terpenoids in *R. tomentosa* young leaves and mature leaves.

**
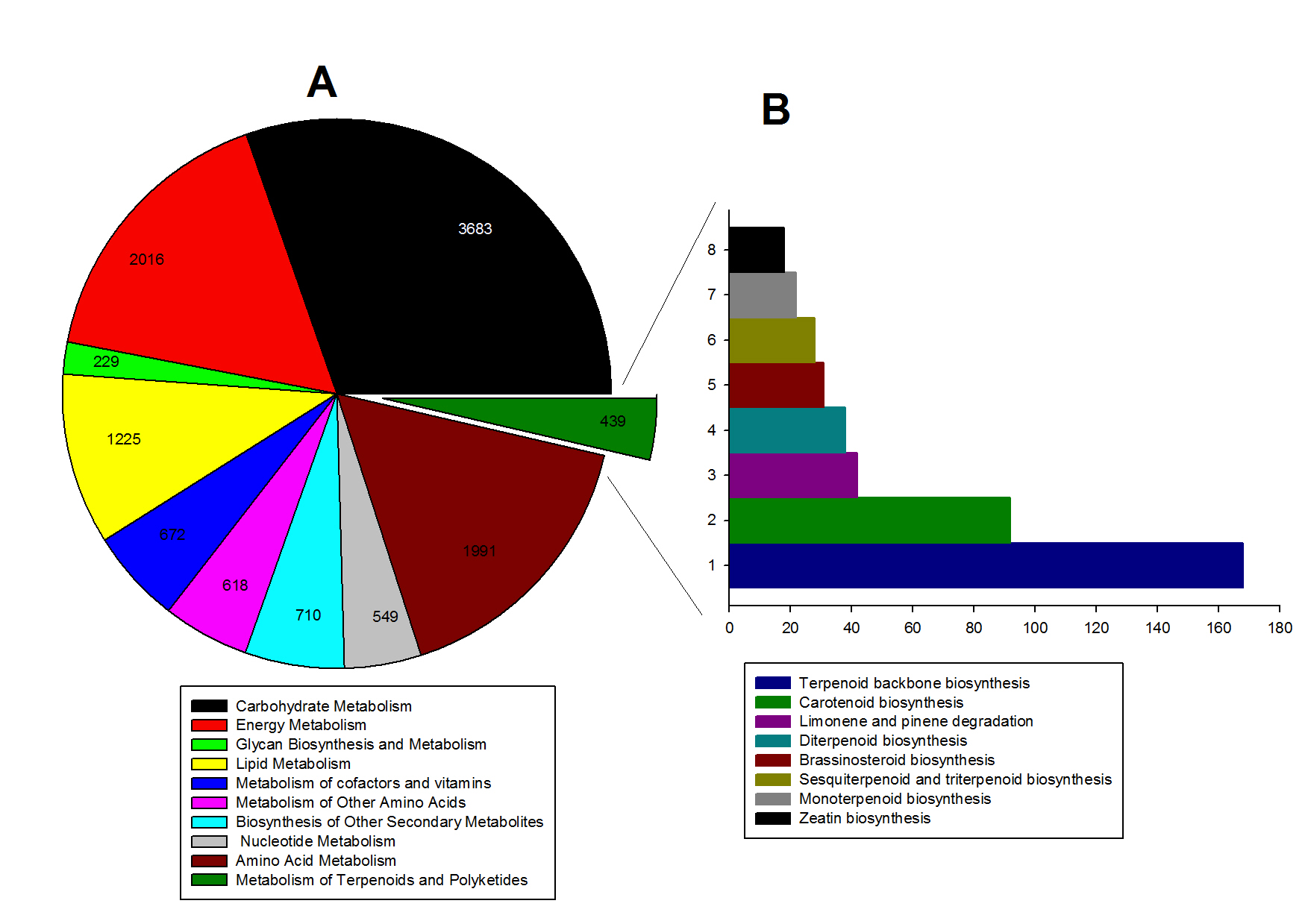
**

**Supplemental Fig. 2. Pathway assignment based on KEGG.** (A) Classification based on metabolism categories; (B) classification based on metabolism of terpenoids and polyketides.
